# The Remote Food Photography Method Is Valid for Assessing Dietary Intake of *Serie A* Football Players

**DOI:** 10.1002/ejsc.70250

**Published:** 2026-10-13

**Authors:** Costanza Gavioli, Florentino Huertas Olmedo, Mathieu Lacome, Rossella Dodi, Mauro Mandorino, Julien Louis

**Affiliations:** ^1^ Performance and Analytics Department Parma Calcio 1913 Parma Italy; ^2^ Doctoral School Catholic University of Valencia “San Vicente Martyr” Valencia Spain; ^3^ Faculty of Physical Education and Sport Sciences Catholic University of Valencia “San Vicente Martyr” Torrent Spain; ^4^ Medical & Performance Department Paris Saint‐Germain Football Club Poissy France; ^5^ Department of Animal Science, Food and Nutrition Università Cattolica del Sacro Cuore Piacenza Italy; ^6^ Research Center for Biochemistry and Sport Nutrition (CRIBENS) Università Cattolica del Sacro Cuore Milan Italy; ^7^ Department of Movement, Human and Health Sciences University of Rome “Foro Italico” Rome Italy; ^8^ Research Institute for Sport and Exercise Sciences Liverpool John Moores University Liverpool UK

## Abstract

To ensure football players meet their nutritional demands, sport nutritionists need to accurately collect and analyse dietary intake. However, dietary assessment methods have limitations. The Remote Food Photography Method (RFPM) is a valid approach, but the validation in real‐life settings requires further investigations. This study aims to evaluate validity, accuracy and agreement of analysing energy and macronutrient intake using the RFPM in *Serie A* football players. Twenty players collected food intake over six nonconsecutive post‐training meals representative of three typical training days. Players independently served themselves food from the club's canteen; plates were then weighed and consequently photographed. Energy, carbohydrate (CHO), protein and fat intake were analysed. RFPM demonstrated a very large validity between weighed and estimated values for CHO (rrmcorr (repeated measure correlation) = 0.87, 95% confidence interval [CI]: 0.82–0.92, *p* < 0.05), protein (rrmcorr = 0.87, 95% CI: 0.81–0.92, *p* < 0.05), fat (rrmcorr = 0.87, 95% CI: 0.81–0.92, *p* < 0.05) and energy intake (rrmcorr = 0.85, 95% CI: 0.78–0.90, *p* < 0.05). Good agreement was observed for CHO −0.83 [−24.41 to 22.75] g, protein 3.23 [−10.87 to 17.33] g, fat 0.85 [−6.96 to 8.66] g and energy 17.27 [−160.41 to 194.95] kcal. There was no systematic bias. Moderate accuracy and acceptable precision, consistent over different training days, were demonstrated for CHO, protein and energy intake. Intra‐operator reliability, performed by repeating 30% of the estimations by the same operator, was good for CHO (CV = 4.7%), protein (CV = 3.6%) and energy (CV = 3.8%) and acceptable for fat intake (CV = 7.8%). The study confirms the RFPM is accurate and reliable for assessing energy and macronutrient intake of elite football players in a real‐training environment.

## Highlights

- Remote Food Photography Method (RFPM) is valid, reproducible and shows good agreement when assessing energy and macronutrient intake in a real‐training environment of *Serie A* football players, across different typical training days.
- The RFPM shows moderate accuracy and acceptable precision in estimating carbohydrate, protein and energy intake, while it is less accurate when assessing fat intake. However, the error is not likely to be significant in daily practice. Accuracy of the method for dietary intake assessment is consistent over all the six nonconsecutive meals. These meals are representative of three different typical training days, in which the quality and quantity of players' food intake varied, to cope with different training demands.
- The RFPM is reproducible when performed by researchers familiar with the food provided.

## 1 Introduction

Football is a highly demanding intermittent sport (Bangsbo 1994), with elite players frequently playing 40 to 60 matches per season and covering around 11–13 km, of which around 1000m are run at speeds above 20 km.h^−1^ (Hulton et al. 2022). This makes training regimes more and more physically demanding too, leading to increased nutritional demands. Indeed, performance, recovery from training and matches and health status are enhanced by adequate nutrition strategies (Collins et al. 2021; Nédélec et al. 2012). Appropriate energy and macronutrient intake can support training and competition, provided that substrate stores meet fuel demands (Thomas et al. 2016), achieving sport‐specific body composition (Mathisen et al. 2023) and avoiding illness and injury (Walsh 2019). Therefore, sports nutritionists should conduct accurate dietary assessment to ensure that athletes adhere to appropriate dietary patterns and meet their energy and macronutrient intake.

Despite this, dietary assessment methods still have evident and well‐known limitations (Bailey 2021). Retrospective (including *24‐hr recall*, food frequency questionnaires and diet histories) and prospective (including food diaries with/without weighed inventory) dietary assessment methods are characterised by substantial random and systematic measurement error (Black 2001). These methods' validity has also been assessed in athletic populations, demonstrating sources of errors present on both the athlete's and sport nutritionist's end (Capling et al. 2017; Martin et al. 2012). In particular, the burden for football players to record dietary intake is exacerbated due to periodised training, more frequent eating episodes (frequent snacking) (Briggs et al. 2015) or total food intake (increased energy requirements) (Bradley et al. 2015). In addition, supplement use and busy training and work schedules increases the likelihood of underreporting, with more and more inaccuracies when assessing sports teams in free‐living conditions (Capling et al. 2017). Moreover, in elite football players daily training and accumulated weekly load have been quantified, showing evidence of periodisation of training load between days within each microcycle (Anderson et al. 2016). Therefore, these data warrant that energy and macronutrient intake be modified and adapted to different training loads and match days (Collins et al. 2021; Anderson et al. 2022), possibly leading to more difficult data collection and monitoring as a consequence of different quality and quantity of food eaten during training days.

A main contributor to bias is the reliance on self‐report without the ability to verify the dietary information. This is due to the fact that maintaining focus or motivation to adhere over long periods is challenging, which typically leads to (a) unintentional under‐reporting of foods (forgetfulness), (b) intentional under‐reporting of foods with negative health images (high‐fat/high‐sugar foods), (c) intentional over‐reporting of foods that are perceived as healthy (fruits, vegetables) and (d) portion size estimation errors (Höchsmann and Martin 2020). Furthermore, the awareness of being assessed may alter eating behaviour (Robinson et al. 2015).

To improve players' reporting accuracy, developing new strategies for energy and food intake estimation is of primary importance. Image‐assisted methods, which rely on photographic images to estimate food intake, are a promising approach preferred by users that can overcome many of the limitations of self‐report. They can reduce user burden and eliminate self‐estimation of portion size, transmitting food image data to researchers in real time or near real time (Gemming et al. 2015; Rollo et al. 2016). A limitation that remains, like with self‐report methods, is intentional and/or unintentional under‐reporting of foods and calorie‐containing beverages due to social desirability or forgetfulness (Zhang et al. 2021). To address this issue, image‐assisted methods for recording dietary intake could be implemented by the use of customized prompts that help participants remember to capture images by sending reminders (text messages, push notifications) around typical mealtimes (Stone and Shiffman 1994). This approach helps in promoting data quality and accuracy, as demonstrated by Martin et al. (2009).

Other than the above‐mentioned advantages of image‐based dietary records in monitoring intake in real‐time (Martin et al. 2009), this method is valid on a population level for analysing nutrient intake, such as within a team or group (Roberts et al. 2024). However, the individual data indicates “poor” validity when assessing energy and macronutrient composition of food, even when the analysis is performed by experienced sport dieticians (Peart et al. 2022; Stables et al. 2021; Costello et al. 2017). These limitations could be even more evident when assessing athletes' food intake (Bradley et al. 2015; Roberts et al. 2024). As a result, these limitations make the identification of athletes with inadequate or poor dietary intake challenging, which can chronically compromise health and performance. In the end, despite demonstrating accuracy in other team sports (Costello et al. 2017), validation studies of image‐based records are lacking among football players. For these reasons, the aim of the present study was to evaluate the validity, accuracy and agreement of analysing energy and macronutrient intake using the Remote Food Photography Method (RFPM) via a free smartphone application in *Serie A* football players in a free‐living environment over six nonconsecutive post‐training meals representative of three different typical training days.

## 2 Methods

### 2.1 Participants

Twenty elite football players belonging to the First team of a club competing in the Italian *Serie A* (Italian first division) were recruited for this study. Before data collection, the lead researcher informed players that the study assessed their energy and macronutrient intake rather than the validity of two dietary assessment methods and the modalities for dietary intake collection were explained.

Ethical approval for the study was obtained from the Catholic University of Valencia “San Vicente Martir” Ethics Committee (UCV/2023‐2024/148).

### 2.2 Experimental Protocol

During lunch, players were required to log their dietary intake across two 3‐day assessment periods over two consecutive weeks, using the RFPM. Before players photographed the food placed on their plate, it was weighed by the second club nutritionist both before and after ad libitum consumption, to account for any leftovers. Both RFPM and weighed food were performed during all lunches, with no randomisation. No dietary advice was provided to the players so that they could eat whatever food they liked and in whatever quantity. If asked, players could also eat more and have a second plate that was weighed and photographed according to the method previously described. No specific macronutrient intake target was set.

The 3 days of data collection were selected as they represented three typical training days: Match Day (MD)‐4 (strength‐oriented session, 4 days before the match), MD‐2 (light training session, technical session, 2 days before the match) and MD‐1 (technical/tactical session, start of the implementation of carbohydrate (CHO) loading, 1 day before the match). Data collection was conducted at the canteen, in the training centre. All food items were provided by the team's canteen as planned in the menu provided by the Club's nutritionist and using the same cutlery and plates.

### 2.3 Dietary Intake Assessment

The food was weighed using a household digital scale sensitive to 1 g (*Gpisen,* Shenzhen, China). The RFPM was implemented via a smartphone application (*WhatsApp* Inc., Menlo Park, CA, USA) with a small description using the canteen's usual portion sizes, cutlery or cups as a size marker to assist with the identification and analysis of foods or items. Before data collection, players were educated about how to take a clear photo of the entire food item or dish before and after consumption. Photographs were to be taken from between 45° and 90° to allow for the judgement of the depth of the food (Roberts et al. 2024). After weighing the food, the photographs were taken and uploaded via the application in near real‐time. Missing photos were not considered in order to test the practical validity of the tool.

Weighed food and RFPM were both analysed using the software *Nutritics* (version 5.5; Swords, Ireland) using the Italian databases available on the software. If a food item was not present in the database, it was added manually in the software, such as for recipes from Club's chef, to have the most accurate information. Since all foods were weighed when already cooked, the weights were converted to the raw portion using the club's conversion factors. They were obtained from weighing 100g of raw food after cooking it. The following conversion factors raw/cooked were used: pasta (short) *1.84, pasta (long) *2, basmati rice *2.74, wholegrain rice *2, white meat, hamburger and fish *0.7, red meat *0.9.

Collection and analysis of weighed food and RFPM were conducted by two different experienced club nutritionists, respectively, with results being blinded from each other. The lead researcher, who was familiar with the type of food served to the players, analysed the photos and recipes. After coding the weights and pictures, a third nutritionist verified all food and drinks inputted in the software so as to add a layer of control.

In order to assess intra‐operator reliability of the method, the lead researcher re‐analysed 30% (*n* = 30) of the pictures 3 months after the first photographs' codification. Inter‐operator reliability was not assessed, since the lead researcher was the only one familiar with the daily dietary practices at the club and including another researcher could have added noise to the results.

## 3 Daily Menu

### 3.1 Match Day−4

After this strength‐oriented session with a high training load, the goal of the post‐training meal was to provide adequate sources of CHO and high‐quality protein. Therefore, the lunch options were low in fibre to meet CHO goals for maximal glycogen synthesis, from a manageable volume of food. It can also help in encouraging intake despite possible gut discomfort and reduced appetite following high‐intensity workouts (Mancin et al. 2025). Food choices were also based on players' preferences.

### 3.2 Match Day−2

After this tactical, low‐training load, the post‐training meal provided high‐fibre (wholegrain cereals, vegetables and beans), lean protein and dietary choices for lower energy requirements. Food choices were made to encourage players to eat more fibre and a greater variety of bioactive compounds.

### 3.3 Match Day−1

On the day prior to the match, even if the training load was lower, the goal was to increase CHO intake to optimise muscle and liver glycogen storage (Collins et al. 2021). Therefore, all foods and drinks provided were rich in CHO and low in fibre. This was a specific nutrition strategy designed to meet the demands of the match, for which players aimed for peak performance. Food choices were white pasta and basmati rice, with an additional amount of CHO also provided as part of the second dish (e.g., potatoes, melon).

All food choices were also based on players' preferences. Details of menus for match day‐4, match day‐2 and match day‐1 can be found in Table 1.

**TABLE 1:** Available food items across assessment periods.

	Match day‐4	Match day‐2	Match day‐1
Week 1	Basmati rice	Brown rice with zucchini and carrots	Basmati rice
	White pasta	Wholegrain pasta	Spaghetti with cherry tomatoes
	Tomato sauce	Tomato sauce	Tomato sauce
	Hamburger	Chicken pieces with soy sauce	Slim beef with potatoes
	Grilled fish	Bean burger	Omelette with vegetables
	Fruit salad	Fruit salad	Fruit salad
Week 2	Basmati rice	Brown rice with vegetables	Basmati rice
	White pasta with cherry tomatoes and zucchini	Wholegrain pasta	Spaghetti with cherry tomatoes
	Tomato sauce	Tomato sauce	Tomato sauce
	Meatballs	Chicken breast, grilled	Ham with melon
	Grilled fish	Bean nuggets	Grilled fish
	Fruit salad	Fruit salad	Fruit salad

*Note:* Vegetables on the buffet: Cucumber, iceberg lettuce, peppers, zucchini and eggplant (grilled), beetroot, cherry tomatoes, spinach, olives, carrots, chickpeas, avocado.

### 3.4 Statistical Analysis

The statistical analysis aimed to evaluate the accuracy, agreement and validity between observed energy intake and energy intake estimated using photos across repeated measures collected over two consecutive weeks, including three distinct days (MD‐4, MD‐2, MD‐1). Moreover, we evaluated intra‐operator reliability after repeating the weighted estimation.

The validity of the two methods (weighed and estimated) was assessed through repeated measures correlation (r_rmcorr_, 95% CI), which evaluated the linear association between the two measures. Correlation magnitudes were classified based on established thresholds: trivial (< 0.1), small (0.1–0.3), moderate (0.3–0.5), large (0.5–0.7), very large (0.7–0.9), nearly perfect (0.9–1.0) and perfect (1.0) (Hopkins 2000). The analysis was conducted for overall energy intake and for individual macronutrients (carbohydrates, proteins, fats).

Accuracy represents how the estimated values of energy and macronutrient intake obtained through the RFPM reflect the actual values measured via food weighing. In this study, accuracy was evaluated through measures of validity and agreement, indicating how close the RFPM estimates were to the true intake values across different meals and training days. To evaluate prediction accuracy, error metrics were calculated: mean absolute error (MAE) to assess the average magnitude of absolute errors and mean absolute percentage error (MAPE) to assess the average percentage deviation between observed and predicted values. These metrics were computed for overall energy intake and macronutrient intake across different days of the week.

Bland‐Altman plots were used to assess agreement between observed and estimated values, focusing on differences in overall energy intake and macronutrients.

Precision denotes the consistency and repeatability of the RFPM estimates when applied under similar conditions. It was assessed through intra‐operator reliability, indicating how much variation existed when the same operator repeated the estimation process. Intra‐operator reliability was investigated by repeating the estimation at 3 months by the same operator. The analysis was performed using intraclass correlation coefficient (ICC) and the typical error of measurement, expressed as a coefficient of variation (CV%). Lower CV values indicate higher reliability between the measurements. CV thresholds were interpreted using thresholds described by Stables et al. (2021): < 2% (excellent), < 5% (good), < 10% (acceptable), > 10% (poor) and > 20% (very poor).

Missing data arising from players missing meals or not providing photographs of their meals, were not considered for analysis. No imputation procedure was applied, because the repeated‐measures correlation can accommodate uneven datasets and does not require the same number of observations for all participants across all measurement occasions. Therefore, all valid observations were retained in the analysis.

The *p*‐value was set at *p* < 0.05. All analyses were performed using Python with relevant libraries from the Anaconda distribution.

## 4 Results

### 4.1 Participants

Twenty elite football players were included in the study (age: 23.8 ± 4.6 years, height: 1.85 ± 0.06 m, weight: 80.3 ± 6.01 kg).

A total of one hundred meals were analysed. Twelve players completed data collection for the six meals considered for the analysis, five players missed one meal because they forgot to take pictures before eating or because of their personal daily training schedules. Three additional players provided data for the last lunch only as they were recruited later in the study, to prevent reduction in sample size because of possible lower compliance of other players throughout data collection.

### 4.2 Energy and Macronutrient Intake

Figure 1 provides an overview of the weighed and estimated (using the RFPM) energy and macronutrient intake for lunch on MD‐4, MD‐2 and MD‐1 over two consecutive weeks.

**FIGURE 1:**
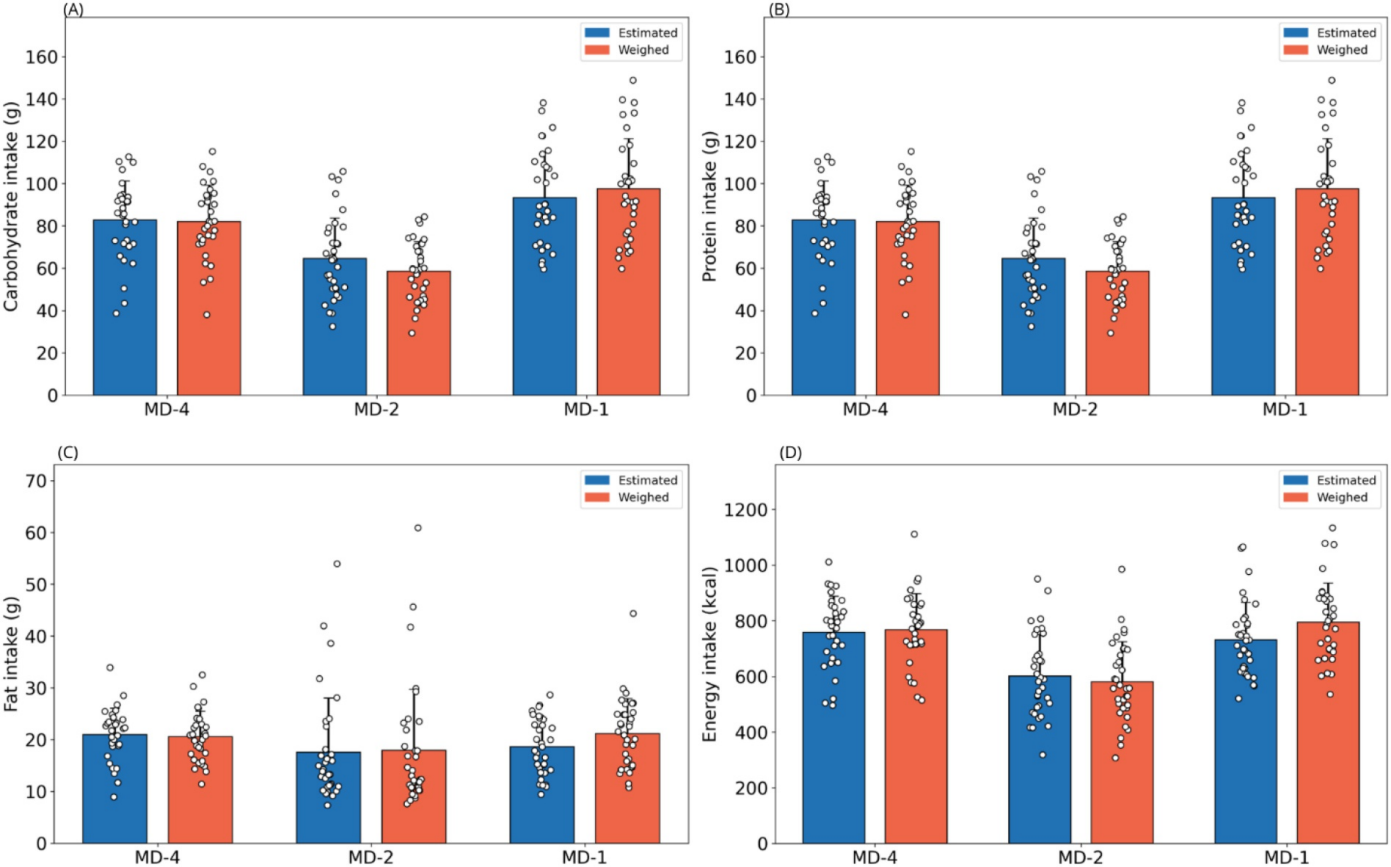
Overview of carbohydrate (A), protein (B), fat (C) and energy intake (D), expressed in g, g/kg BW and kcal, respectively, for Match Day (MD)‐4, MD‐2 and MD‐1 over two consecutive weeks. Data is presented as mean + standard deviation and individual data points represent every single player's meal. Estimated and weighed carbohydrate, protein, fat and energy intake are presented in blue and red, respectively.

Mean estimated CHO intake [mean ± SD] (Figure 1A) over two consecutive weeks was 82.8 ± 18.4 g on MD‐4, 64.6 ± 19.1 g on MD‐2 and 93.3 ± 21.5 g on MD‐1. These values are equivalent to 1.0 g/kg BW, 0.8 g/kg BW and 1.2 g/kg BW, respectively. Mean weighed CHO intake over the 2 weeks was 82.1 ± 17.2 g on MD‐4, 58.6 ± 14.0 g on MD‐2 and 97.6 ± 23.6 g on MD‐1. These ranges are equivalent to 1.0 g/kg BW, 0.7 g/kg BW and 1.2 g/kg BW, respectively.

On the other hand, mean estimated protein intake (Figure 1B) over the 2 weeks was 59.7 ± 15.3 g on MD‐4, 46.3 ± 11.4 g on MD‐2 and 47.8 ± 10.3 g on MD‐1, equivalent to 0.7 g/kg BW, 0.6 g/kg BW and 0.6 g/kg BW, respectively. Weighed protein intake on both weeks was 63.7 ± 15.5 g on MD‐4, 46.3 ± 11.9 g on MD‐2 and 53.5 ± 10.2 g on MD‐1, equivalent to 0.8 g/kg BW, 0.6 g/kg BW and 0.7 g/kg BW, respectively.

Mean estimated and weighed fat intake (Figure 1C) over the 2 weeks were quite similar in different days, ranging from [min‐max] 7.3–54.0 g and 7.6–60.9 g, equivalent to 0.20–0.27 g/kg BW and 0.24–0.30 g/kg BW, respectively.

Mean estimated and weighed energy intake (Figure 1D) along the two weeks of dietary assessment were 318–1066 kcal and 308–1134 kcal, respectively.

The repeated measures correlation analysis demonstrated a very large positive correlation between weighed and estimated values for CHO (*r*_rmcorr_ = 0.87, 95% CI: 0.82–0.92, *p* < 0.05) (Figure 2A), protein (*r*_rmcorr_ = 0.87, 95% CI: 0.81–0.92, *p* < 0.05) (Figure 2B), fat (*r*_rmcorr_ = 0.87, 95% CI: 0.81–0.92, *p* < 0.05) (Figure 2C) and energy intake (*r*_rmcorr_ = 0.85, 95% CI: 0.78–0.90, *p* < 0.05) (Figure 2D).

**FIGURE 2:**
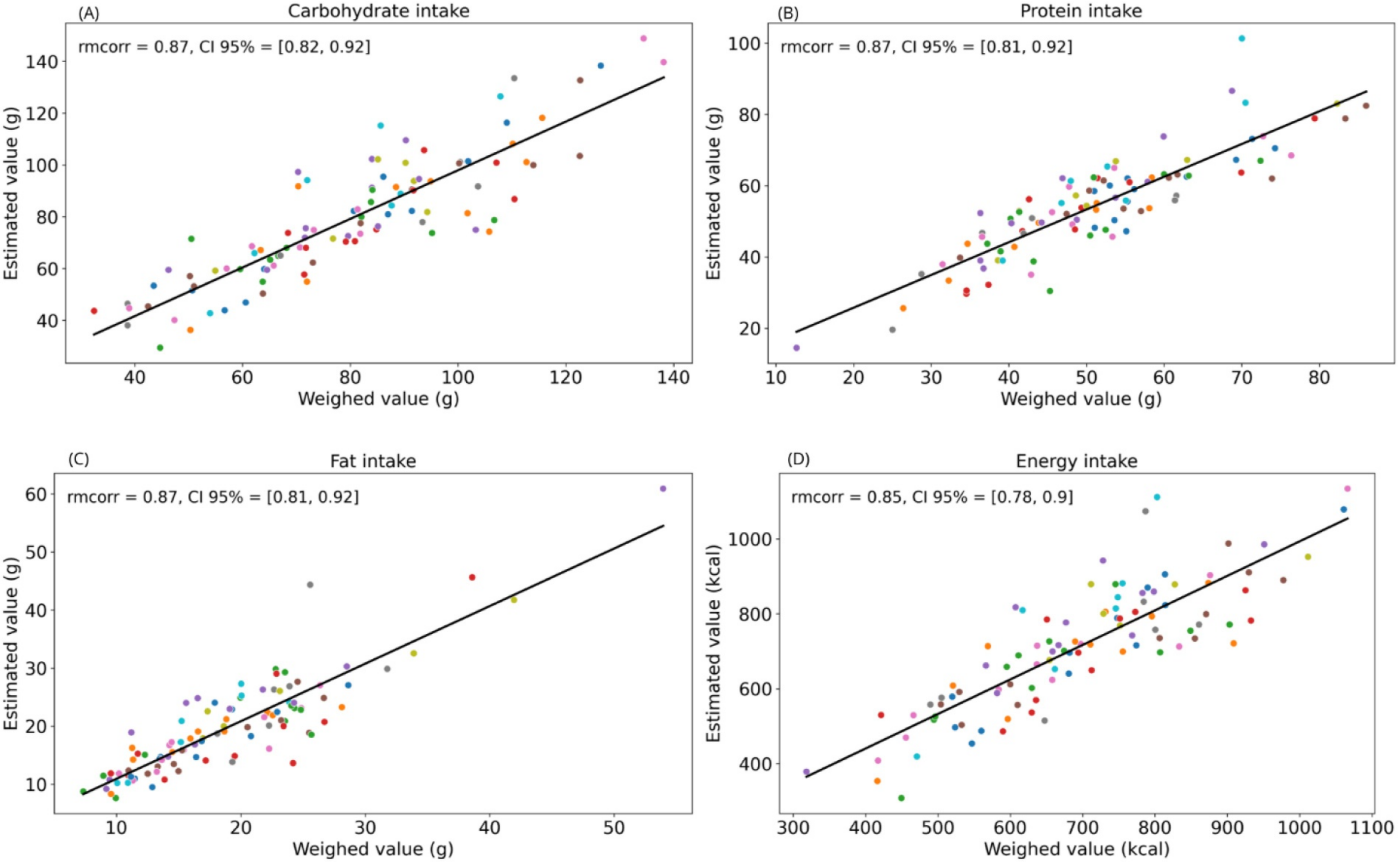
Relationship between estimated value and weighed value for carbohydrate (A), protein (B), fat (C) and energy intake (D). rmcorr = repeated measure correlation, CI = Confidence Interval. The individual data points and colours represent single players' meals analysed.

The MAE and MAPE values for the macronutrient intake, analysed across different days of the week, are presented in Table 2. The highest MAPE for CHO intake of 17.2% is recorded on MD‐2, while the highest for protein and fat intake (13.0% and 17.1% respectively), are recorded on MD‐1. The highest MAPE for energy intake is 10.6%, in MD‐2.

**TABLE 2:** Mean absolute error (MAE) and mean absolute percentage error (MAPE) calculated for carbohydrate, protein, fat and total energy intake in relation to the day of the week.

	Carbohydrate		Protein		Fat		Total energy	
MD	MAE (g)	MAPE (%)	MAE (g)	MAPE (%)	MAE (g)	MAPE (%)	MAE (kcal)	MAPE (%)
MD‐4	8.7	10.7	6.9	11.0	2.8	14.8	77.3	10.0
MD‐2	9.4	17.2	4.7	10.6	2.1	12.2	55.5	10.6
MD‐1	9.3	9.3	6.9	13.0	3.7	17.1	84.4	10.5

The results of the Bland‐Altman analyses are shown in Figure 3. The mean difference between weighed and estimated values for CHO is −0.83 [−24.41 to 22.75] g Figure 3A, 3.23 [−10.87 to 17.33] g for protein Figure 3B, 0.85 [−6.96 to 8.66] g for fat (Figure 3C) and 17.27 [−160.41 to 194.95] kcal for energy intake (Figure 3D). Accordingly, there was no systematic bias.

**FIGURE 3:**
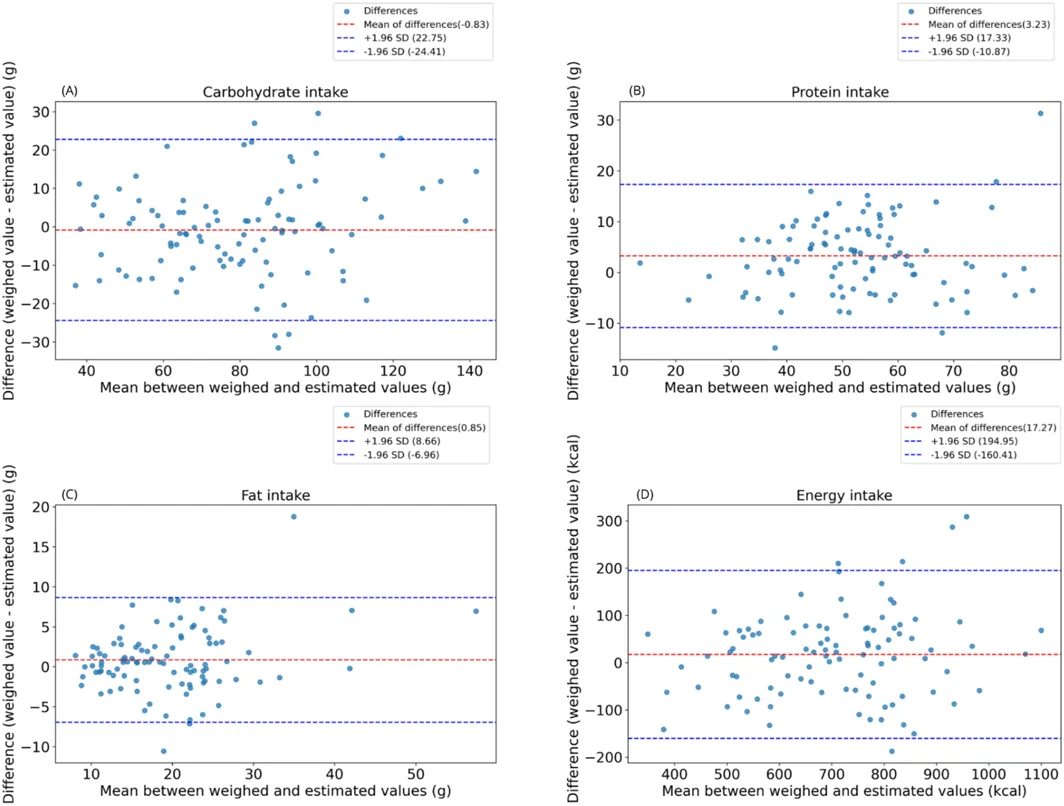
Bland‐Altman analysis for carbohydrate (A), protein (B), fat (C) and energy (D) intake. The data points represent single players' meals analysed. SD = Standard deviation.

The intra‐operator reliability (Table 3) was generally good for the estimation of CHO (CV = 4.72%) and protein intake (CV = 3.60%), acceptable for fat intake (CV = 7.78%) and good for energy intake (CV = 3.81%).

**TABLE 3:** Intra‐operator reliability calculated for carbohydrate, protein, fat and energy intake.

Variable	ICC	95% CI	CV (%)
Carbohydrate	0.94	0.87–0.98	4.72
Protein	0.98	0.96–0.99	3.60
Fat	0.92	0.75–0.97	7.78
Energy	0.97	0.94–0.99	3.81

Abbreviations: CI: Confidence Interval, CV: Coefficient of Variation, ICC: Intraclass Correlation Coefficient.

## 5 Discussion

The purpose of the present study was to examine the ecological validity of the RFPM for measuring energy and macronutrient intake in *Serie A* football players in a real‐training environment over six nonconsecutive post‐training meals representative of three different typical training days, compared to weighted food intake. A very large validity of the RFPM was observed for both energy and macronutrient intake, compared with the weighed values. Furthermore, the lack of systematic bias between RFPM and the weighed food method for energy and macronutrient intake suggests the RFPM presents good agreement at a group level, making it a useful tool to be used in an ecological setting. In addition, intra‐operator reliability was acceptable for macronutrient and energy intake, as inferred through CV values. The validity and reliability of RFPM confirm it as an accurate and precise method with acceptable estimation errors for assessing the actual energy and macronutrient intake of *Serie A* football players in a real‐training environment.

In the present study, the relationship between the gold standard method to analyse dietary intake (weighed food) and RFPM was “very large” both for macronutrient and energy intake. This is in line with previous studies in athletic populations (Capling et al. 2017; Roberts et al. 2024; Costello et al. 2017). On the contrary, Stables et al. (2021) reported “poor” or “very poor” validity of the RFPM in estimating CHO intake, “acceptable” to “poor” for protein intake and “acceptable” for fat intake. This highlights the importance of a priori food knowledge when using the RFPM. In the study by Stables et al. researchers who performed the dietary assessment had no prior knowledge of the food provided, while in our study the lead researcher had prior knowledge of the type and quality of food provided, recipes and usual portion sizes, but not the quantity of the food, likely leading to an overall better estimation.

Upon initial interpretation, agreement represented by low levels of bias warrants the practical use of the RFPM for the measurement of energy and macronutrient intake at a group level, as demonstrated by other studies (Roberts et al. 2024). However, we evaluated agreement only for group‐level comparisons, which is generally acceptable, while other studies reported that individual agreement is generally weaker (Capling et al. 2017; Roberts et al. 2024). In the Bland‐Altman analysis for protein intake, a higher mean bias was observed compared to other macronutrients, indicating a systematic overestimation by the RFPM compared to the weighed method. However, the dispersion within the limits of agreement was relatively narrow, suggesting moderate accuracy. In conclusion, there is no visual trend for under‐ or overestimation in the Bland‐Altman plot for all the macronutrients and energy intake.

Considering accuracy, the MAPE of estimation for macronutrient and energy intake changes across the different days of the week. The RFPM showed a moderate level of accuracy in estimating CHO, protein and energy intake across the 3 days and a generally acceptable level of precision. The method is less accurate in the estimation of fat. For CHO, MAE for estimation is consistent across different days of dietary assessment, so it is reasonable to suggest that the RFPM is not biased by under or overreporting, considering that total CHO intake was likely different on MD‐4, MD‐2 and MD‐1 due to differences in training load and meal composition. The lowest MAPE for CHO estimation was observed on MD‐1, which may reflect differences in meal composition that facilitated easier estimation. Similarly for the assessment of energy intake, it can be assumed that the estimation was precise even for higher intakes because MAE or MAPE did not increase on MD‐4, for which energy intake was likely greater than for the other days. This is in line with previous studies (Martin et al. 2009; Flegal et al. 2020) showing that the mean difference in estimating total energy intake was stable over different levels of intake. On the other hand, MAPE for fat intake was higher when energy intake was greater, as reported in other studies (Capling et al. 2017; Roberts et al. 2024). The periodization of training load between days within the weekly microcycle could influence energy and macronutrient demands, leading to differences in the quality and quantity of food ingested, which could make dietary assessment more difficult. However, based on the present data, we can say that the RFPM is moderately accurate in the assessment of energy and macronutrient intake independently of training days.

Estimating errors can be a consequence of players' forgetting to take pictures of food they were eating or misreporting because of social expectations or the nature of the testing environment. In addition, estimating errors could also be caused by the researcher's inability to code portion sizes from the pictures received. For example, the underestimation of fat intake visible from outliers in the Bland‐Altman plot could be explained by players adding extra virgin olive oil to their plates that was not always detectable from pictures. On the other hand, overestimation could be due to players forgetting to take pictures of leftovers or inability to properly codify from pictures. Despite this, it is also important to consider whether under‐ or overestimation of macronutrient intake is clinically significant (Hamilton and Stamey 2007; García‐Rovés et al. 2014). In this case, considering the highest MAE for each macronutrient, the error of estimation for CHO intake on MD‐2 was 0.12 g/kg BW, 0.09 g/kg BW for protein intake on MD‐1, 0.05 g/kg BW for fat intake on MD‐1 and 84.4 kcal for energy intake. These quantities are unlikely to be meaningful when assessing macronutrients and energy intake of a specific meal. It is noteworthy that the daily magnitude of the estimation could be more important considering that athletes are likely prone to more frequent eating episodes throughout the day (Briggs et al. 2015).

This is the first study to make a qualitative description of a post‐training meal on different typical training days in *Serie A* elite football players. Few studies have investigated the food sources of the nutrients ingested by football players (Anderson et al. 2017). While quantitative description is necessary for describing macronutrient and energy intake (Brinkmans et al. 2019; Foo et al. 2025), the qualitative approach helps us understand the behavioural factors influencing food choices, such as personal preferences, club context, cultural habits and ethnicity, perceived body composition (Braakhuis et al. 2003), together with a deeper understanding of the athletes' attitudes, beliefs and experiences regarding post‐training nutrition. This is particularly relevant in elite football, where ensuring better recovery is one of the primary nutritional goals and can help identify barriers to optimal intake and inform more effective, individualized nutritional strategies.

While the results of this validation study indicate the RFPM may be a valid and accurate tool for analysing energy and macronutrient intake in elite football players, it is not without limitations. The data was collected on a small sample size, for a short period of time and assessed during one meal (lunch only). Therefore, we cannot conclude on the validity and accuracy of the method for other meals or snacks of the day, also considering that previous studies have shown that breakfast and snacks are more prone to overestimation of dietary intake because of the large variety of food and drink options (Stables et al. 2021). Moreover, we are conscious that some of the MAE and MAPE observed effects did not reach statistical significance, which may be partly related to the limited sample size used in this study. This highlights that the findings should be interpreted cautiously and that further research with larger samples is warranted to confirm the observed results and provide more robust evidence regarding the accuracy and validity of the proposed method. In addition, we did not test inter‐practitioner reliability, which has been reported as generally “poor” in other studies when practitioners had no prior knowledge of the food typically consumed (Anderson et al. 2022; Roberts et al. 2024). Our decision to have only the lead nutritionist estimating dietary intake using the RFPM was justified by the a priori knowledge of the cutlery, portion sizes and recipes typically utilized at the club canteen. Therefore, we can be confident that the RFPM can be used by sport nutritionists internal to clubs, where repeated weighing of food can be time‐consuming and burdensome, to monitor dietary intake and ensure players meet their energy and macronutrient needs. This highlights the importance of the team's sport nutritionist in assessing players' dietary intake, when compared to external consultants.

According to the present study, the RFPM may be a feasible alternative to weighted food records for monitoring dietary intake in elite football players. Although individual meal estimates were associated with a moderate but acceptable error, the method presented no systematic bias, a very large validity and high intra‐operator reliability. The RFPM could therefore facilitate the routine monitoring of energy and macronutrient intake and help sport nutritionists identify players who may not meet individualized nutritional targets through early screening. From a performance perspective, this is of particular importance for CHO intake during the days preceding competition: identifying players who are eating enough CHO, that is who change their intake according to the training load, from those who do not meet individual targets.

## 6 Conclusion

The present study demonstrates that the RFPM is valid for monitoring energy and macronutrient intake in elite football players from a *Serie A* team, across different training days. When compared to the gold standard weighed food method, RFPM is precise and accurate, especially at group level. When assessing single macronutrient or energy intake, moderate accuracy has been shown for CHO, protein and energy intake. Mean absolute error is consistent and acceptable over different levels of CHO, protein and energy intakes, making RFPM accurate in estimating these variables and accuracy is consistent across three different typical training days. On the other hand, the method is less accurate in assessing fat intake, however, the error is unlikely to be meaningful when assessing daily macronutrient intake. RFPM is also reproducible when performed by researchers familiar with the food provided. This makes the RFPM a useful tool for club nutritionists to monitor football players' daily food intake.

## Funding

The authors have nothing to report.

## Ethics Statement

The study was conducted according to the Declaration of Helsinki guidelines and approved by the Catholic University of Valencia San Vicente Martir Ethical Committee (UCV/2023‐2024/148).

## Conflicts of Interest

The authors declare no conflicts of interest.

## Data Availability

The data that support the findings of this study are available from the corresponding author upon reasonable request.
